# Congenital syphilis in the twenty-first century: an area-based study

**DOI:** 10.1007/s00431-022-04703-5

**Published:** 2022-11-14

**Authors:** Serena Salomè, Maria Donata Cambriglia, Sara Maria Scarano, Eleonora Capone, Ivy Betts, Daniela Pacella, Matilde Sansone, Laura Letizia Mazzarelli, Andrea Lo Vecchio, Giusy Ranucci, Geremia Zito Marinosci, Letizia Capasso, Paola Salvatore, Francesco Raimondi

**Affiliations:** 1 Department of Translational Medical Sciences – Division of Neonatology, University of Naples Federico II, Via Pansini 5, 80131 Naples, Italy; 2 Department of Translational Medical Sciences, Pediatric Infectious Disease Unit, University of Naples Federico II, Naples, Italy; 3 Department of Public Health, University of Naples Federico II, Naples, Italy; 4 Department of Translational Medical Sciences – Section of Obstetrician and Gynecologist, University of Naples Federico II, Naples, Italy; 5 Department of Pediatrics, Santobono-Pausilipon Children’s Hospital, Naples, Italy; 6 Service of Anesthesia and Critical Care, Department of Anesthesia and Critical Care, Santobono-Pausilipon Children’s Hospital, Naples, Italy; 7 Department of Molecular Medicine and Medical Biotechnology, University of Naples Federico II, Naples, Italy

**Keywords:** Congenital syphilis, Maternal syphilis, Syphilis epidemiology, Surveillance

## Abstract

The resurgence of syphilis and subsequent risk for newborns has been described worldwide; however, European data on this congenital infection is lacking. We report the activity of a multidisciplinary specialized unit assisting a large area in the Southern Italy. A retrospective cohort study has been conducted at the Perinatal and Pediatric Infectious Diseases Units of the Federico II University of Naples, enrolling all newborns and children referred from January 2010 to June 2022 exposed to *Treponema pallidum* in utero and/or congenitally infected. A total of 323 patients were included in the analysis. Twenty (6.2%) received a diagnosis of confirmed congenital syphilis (CS) and one died. Fifteen CS cases had typical clinical features. Since 2017, the number of referred neonates tripled while the rate of late maternal diagnoses did not significantly differ. When compared with mothers of exposed infants, mothers of CS cases were younger (25 ± 7.2 vs 29.9 ± 6 years, *p* = *0.041*), had less previous pregnancies (0.64 vs 1.11, *p* = *0.044*), and received a diagnosis of syphilis at a later stage of pregnancy (86% vs 20%, from third trimester or later on; *p* < 0.001). Appropriate maternal therapy was protective against vertical transmission (− 1.2; − 1.4, − 1 95% CI; *p* < 0.001). Paternal syphilis status was known in 36% of cases.

*Conclusion*: CS has still a significant impact. Prevention should be implemented towards specific maternal risk profiles. A specialized unit is the preferable model to improve surveillance and healthcare for this neglected population.

**Table:** 

**What is Known:** *• The resurgence of syphilis and subsequent risk for newborns has been described worldwide.* *• European data on this congenital infection is lacking.*	
**What is New:** *• Congenital syphilis has a significant impact still in Europe and prevention should be implemented towards specific maternal risk profiles. * *• A specialized unit is the preferable model to improve surveillance and healthcare for this neglected population.*

**Supplementary Information:**

The online version contains supplementary material available at 10.1007/s00431-022-04703-5.

## Introduction

Congenital syphilis (CS) is a life-threatening infection caused by mother-to-child transmission (MTCT) of *Treponema pallidum* [1]. Syphilis during pregnancy can lead to miscarriage, perinatal death, prematurity, intrauterine growth retardation, or symptomatic children [2–4]. Morbidity and mortality risks during the perinatal period are estimated at 33.6% and 6.5%, respectively [5]. Two-thirds of infected children are asymptomatic at birth although, if left untreated, they can develop symptoms of disease months to years later [6] and the burden of permanent sequelae is significant [7].

CS is a preventable and treatable infection by universal prenatal screening and maternal treatment with penicillin. In 2007, the World Health Organization (WHO) launched the global initiative for its global eradication [8] establishing a desirable rate under 0.5 cases per 100,000 live births. Since then, however, several authors reported a significant number of infants diagnosed with CS, especially in China [9] and the Americas [7, 10–12]. This may be linked to both the general resurgence of syphilis reported even in high-income countries [11, 13] and the uncertain efficacy of national CS surveillance programs, when present.

In Europe, national CS documents show a clear unbalance with rates varying across countries ranging from 0.1 to 39.8 cases per 100,000 live births [14]. Unfortunately, limited information is available about the outcome of these infants. We believe that the issue requires more attention and propose the model of a Specialized Perinatal Infection Unit (SPIU). This paper summarizes the data from our SPIU related to CS offering some considerations on the possible advantages of an area-based approach.

## Patients and methods

### Study protocol and patient cohort

This is a single-center, retrospective cohort study conducted at the SPIU of the Federico II University of Naples, enrolling all newborns and children referred from January 2010 to June 2022 who were exposed in utero to *T. pallidum* and/or congenitally infected. The multidisciplinary team includes Neonatology, Maternal Fetal Medicine, and Pediatric Infectious Diseases specialists taking care of the mother and infant dyad referred for vertically transmitted infections for the whole Campania Region (618,411 live births from 2010 to June 2022).

Maternal infection was defined according to national and international guidelines [15, 16]. Data was collected regarding specific serological evaluations, time of diagnosis of syphilis, stage of disease in pregnancy, maternal therapy carried out in dosages and times, and adequacy of treatment. Moreover, we included demographic and social risk factors, such as age, country of origin, marital status, drug abuse, and co-infection by other sexually transmitted agents.

Mothers were defined as having received an adequate treatment if they received a penicillin regimen according to their stage of disease, if treatment was administered more than 4 weeks before delivery, and if documented test results showed evidence of a fourfold decrease of non-treponemal titer.

### Laboratory evaluation

Serum samples were collected at admission and examined with the following tests:

- Non-treponemal test (NTT) such as the rapid plasma reagin (RPR) test
- Treponemal test (TT) such as *T. pallidum* hemagglutination test (TPHA)
- Specific enzyme-linked immunosorbent assay (ELISA) anti-*T. pallidum* IgG and IgM antibody tests

For the purpose of comparison, mothers’ serum samples were also collected at infant admission and examined with the same tests.

Cerebrospinal fluid (CSF) examination was performed for infants with confirmed CS and/or high risk of CS. Results were considered abnormal if CSF white blood cell count was > 25/ml, CSF protein was > 400 mg/dL, and/or an NTT [17, 18] was reactive.

Since 2018, polymerase chain reaction (PCR) testing was performed at first evaluation using blood samples.

### Instrumental evaluation

Infants underwent an instrumental assessment of the central nervous system, fundus oculi examination, and hearing function evaluation within the first month of life. Cranial ultrasonography (CUS) was performed by an experienced neonatologist, who was blinded to the clinical data, using the Philips HD11 ultrasound imaging platform with an 8.5–12.4-MHz transducers (microconvex and phased array transducers). The fundus oculi examination was performed by a pediatric ophthalmologist skilled in congenital infections. The hearing function was evaluated by transitory evoked otoacoustic emissions (TEOAE) at birth and by automatic auditory brainstem response (ABR) after 3 months of life if the previous evaluation was not normal. In addition, we performed an echocardiogram and abdominal ultrasound, and high-risk newborns underwent a long bone X-ray.

At-risk patients were classified according to the Centers for Diseases Control and Prevention (CDC) criteria [19].

All infants born to syphilis-seropositive women were evaluated for clinical evidence (e.g., skin rash, hepatosplenomegaly, cholestatic jaundice), and laboratory abnormalities (e.g., elevated liver transaminase, elevated conjugated bilirubin, anemia, thrombocytopenia, leukocytosis, elevated C reactive protein, proteinuria). Long bone radiographs were determined to be consistent with CS if osteochondritis or perichondritis were highlighted.

The diagnosis of CS was defined by the presence of one or more of the following criteria:

- Child had symptoms suggestive of the diagnosis;
- Titer of the neonatal non-treponemal test was fourfold higher than the maternal test at the time of delivery;
- IgM were positive at the first evaluation;
- Neonatal PCR test was positive;
- Persistent positivity of specific serology after 12 months of life was detected.

Conversely, infants were considered only exposed in utero to *T. pallidum* but not infected in the case of negative treponemal and non-treponemal antibody titres, previous evidenced at birth, at the end of an adequate follow-up at 12–18 months.

All infected infants and children were treated with intravenous aqueous penicillin G, at the recommended dosage for 10 days, except for patients diagnosed with neurosyphilis who received prolonged therapy for 14 days.

Those who cannot be diagnosed of confirmed CS at birth were evaluated according to national and international guidelines to define the probability of congenital infection and the subsequent appropriate therapeutic strategy in relation to the risk of maternal–fetal transmission [15, 16]. They received preventive treatment with intravenous aqueous penicillin G, at the recommended dosage for 10 days, or with a single dose of intramuscular benzathine penicillin in case of unreliable IV therapy (such as parental deny to hospitalization) or low risk but possible lack of follow-up.

These infants were followed up until 12 or 18 months of age when the diagnosis of CS was either ruled out or confirmed. Furthermore, after their initial evaluation, patients underwent serological checks every three months until 18 months if received any type of treatment at birth, and until 12 months for all other patients to evaluate the time to negativization of Treponemal and non-Treponemal tests. In case of CS, once diagnosed, the outcomes of the infection acquired in utero were defined at the end of evaluation period, ideally until 6 years of age.

The inclusion criteria comprised of maternal history of syphilis, a positive treponemal and/or non-treponemal serology, and a regularly and complete follow-up including results of the laboratory, instrumental, and clinical investigations carried out in the following 12–18 months. Exclusion criteria were parental denial to participate, and infants who did not complete the follow-up.

Official data on regional livebirths published by the Italian National Institute for Statistics were used to calculate CS incidence rates.

### Statistical analysis

Numerical variables were described using mean ± standard deviation (SD) or median with range [interquartile range (IQR)], while categorical variables were summarized using absolute frequencies and percentages. Between-group differences were assessed accordingly using the Student’s *T* test for independent samples or the Mann–Whitney *U* tests when appropriate. The chi-square test or the Fisher’s exact test were used to investigate categorical variables. Associations between the considered factors and the outcomes of interest were analyzed using either multiple logistic regression models, estimating odds ratios (ORs) with the corresponding 95% confidence intervals (95% C.I.), or multiple linear regression models. All analyses were conducted using a two-tailed significance level of 0.05. All data analyses were performed using the R statistical software version 4.0.3 (R Core Team 2018).

## Results

### Population characteristics

**Fig. 1:**
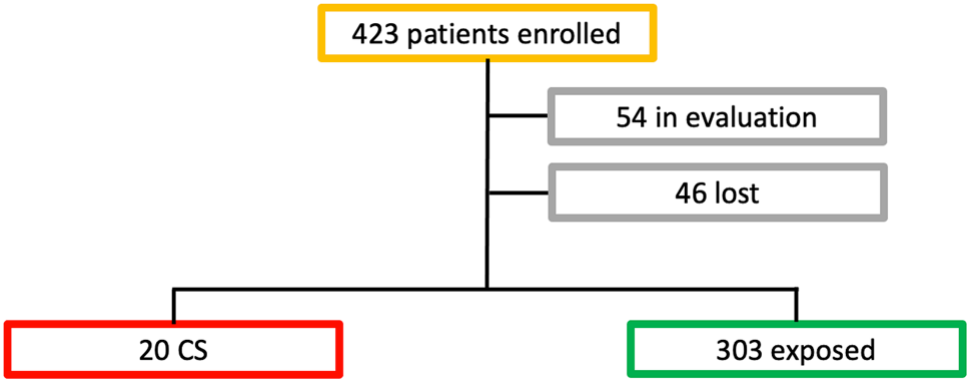
Study population

**Fig. 2:**
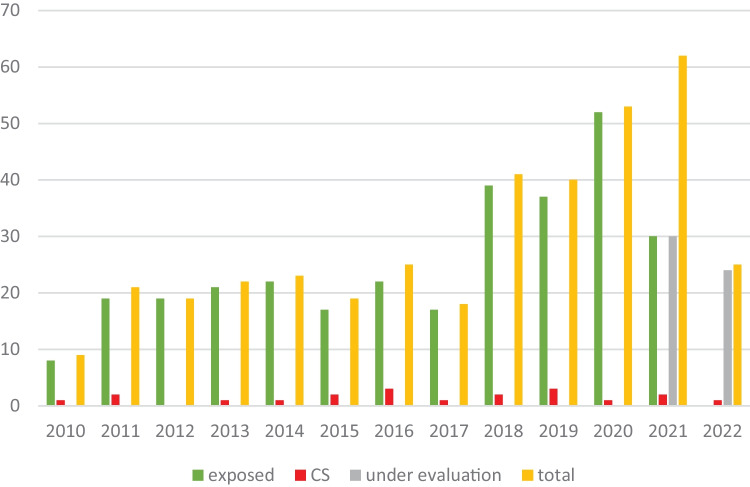
Distribution per year

From January 2010 to June 2022, we collected data from 423 children, including infants born to mothers who had seropositive syphilis or infants diagnosed with CS without a previous known history of maternal infection. According to the established inclusion and exclusion criteria, 46 patients were lost to follow-up and other 54 are still in follow-up and were not included in the present study. The flow chart of the study population is shown in Fig. 1 and the distribution per year in Fig. 2.

### Characteristics of exposed and infected patients

Of the 323 included patients, 20 (6.2%) were diagnosed with CS, while the others were only exposed to infection during intrauterine life, but not considered infected at the end of the evaluation period. Whenever CS was clinically suspected, the patient subsequently underwent a full serological assessment. One neonate, whose mother had syphilis diagnosed in pregnancy, underwent lab tests before any abnormality was detected at physical examination.

Clinical, serological, and instrumental characteristics of infected children are described in Table 1.

**Table 1:** Clinical, serological, and instrumental characteristics of infected children

	**Sex**	**BW (g)**	**GA**	**Access to prenatal care**	**Maternal treatment during pregnancy**	**CS diagnosis: serologic tests and others investigations**	**CS diagnosis: clinical features**	**Long bone X-ray**	**CSF**	**Timing RPR negativization**	**Outcome**
1	M	2500	34	Yes	Untreated	RPR fourfold > than mother’s titer, CSF +	Asymptomatic	Normal	NTT +	3 months	Delayed acquisition of developmental milestones but normal later
2	M	3220	39	Yes	Untreated	Symptoms, RPR fourfold > than mother’s titer	Cutaneous lesions	Normal	Normal	6 months	Normal neurological outcome
3	F	2620	40	Yes	Untreated	Symptoms, RPR fourfold > than mother’s titer, CSF +	Cutaneous lesions	Normal	Protein 323 mg/dl, WBC 90/mm^3^ NTT +	6 months	Normal neurological outcome
4	M	2000	32	Yes	Unknown	RPR fourfold > than mother’s titer, CSF +	Asymptomatic	Normal	Protein 172 mg/dl, NTT +	3 months	Normal neurological outcome
5	M	1680	33	Yes	Treated but unresponsive (impending delivery prevented a second penicillin course)	Symptoms, RPR fourfold > than mother’s titer, CSF +	Jaundice, hepatosplenomegaly, diffuse oedema, thrombocytopenia, petechiae, anemia, palmar-plantar lamellar desquamation, respiratory distress	Normal	NTT +	6 months	Normal neurological outcome
6	F	3000	38	Yes	Untreated	IgM reactivity	Asymptomatic	Normal	Normal	9 months	Normal neurological outcome
7	M	2970	39	Yes	Untreated	IgM reactivity	Sepsis	Normal	Not practiced	3 months	Normal neurological outcome
8	F	2450	34	Yes	Untreated	Symptoms, RPR fourfold > than mother’s titer, IgM reactivity	Cardiorespiratory depression, cutaneous lesions	Normal	Normal	9 months	Autism spectrum syndrome
9	M	2470	39	Yes	Untreated	Symptoms, RPR fourfold > than mother’s titer	Cutaneous bullous lesions, jaundice, hepatosplenomegaly, anemia	Normal	Normal	9 months	Attention-deficit hyperactivity disorder
10	M	2340	34	Yes	Untreated	IgM reactivity, CSF +	Asymptomatic	Normal	NTT +	6 months	Normal neurological outcome
11	M	4140	38	Yes	Untreated	Symptoms, IgM reactivity, CSF +	Fever, cutaneous maculopapular lesions, anemia	Normal	NTT +	12 months	Normal neurological outcome
12	F	2130	39	Yes	Untreated	Symptoms, RPR fourfold > than mother’s titer, IgM reactivity	Fever, cutaneous maculopapular lesions, pneumonia, osteitis	Abnormal	Not practiced	9 months	Normal neurological outcome
13	F	3350	41	Yes	Untreated	Symptoms, RPR fourfold > than mother’s titer, IgM reactivity, CSF +	Cutaneous lesions, periostitis and osteitis of radius and ulna, anemia	Abnormal	NTT +	12 months	Normal neurological outcome
14	M	3940	42	Yes	Untreated	Symptoms, IgM reactivity, CSF +	Saber shins, Hutchinson teeth, poor growth	Abnormal	NTT +	Still positive at 20 months	Delayed acquisition of developmental milestones but normal later; dental anomalies
15	M	4160	40	Yes	Untreated	Symptoms	Chorioretinal scar	Normal	Normal	4 months	Normal neurological outcome, normal visual function
16	F	2490	36	Yes	Untreated	Symptoms, IgM reactivity, CSF +	Hepatosplenomegaly, thrombocytopenia, coagulation disorder, anemia, seizures on EEG, periostitis and osteitis of tibia	Abnormal	Protein 167 mg/dl, WBC 113/mm^3^ NTT +	4 months	Normal neurological outcome, persistent radiological abnormalities without clinical signs
17	M	1600	31	Yes	Untreated	Symptoms	Hydrops, cutaneous palmar-plantar bullous lesions, petechiae, thrombocytopenia, anemia, labial fissures, periostitis and osteitis of radius, homer, fibula, tibia, femur	Abnormal	Normal	4 months	Delayed acquisition of developmental milestones (even if corrected for gestational age)
18	M	3300	38	Yes	Untreated	Symptoms, RPR fourfold > than mother’s titer, CFS +	Multi-organ failure, liver failure, multiple osteochondritis	Abnormal	NTT +	Still positive at death	Death
19	F	2900	38	Yes	Untreated	Symptoms, RPR fourfold > than mother’s titer, CFS +	Respiratory distress and respiratory failure, cutaneous rash, hypothyroidism, anemia, lymphadenopathy, multiple osteochondritis, hepatosplenomegaly	Abnormal	NTT +	Still positive at 4 months	Mild developmental delay
20	M	3100	42	Yes	Untreated	Symptoms	Osteomyelitis and multiple osteochondritis	Abnormal	Normal	2 months	Unknown

Basic characteristics of confirmed CS and exposed in utero cases are shown in Table 2.

**Table 2:** Basic characteristic of confirmed CS and exposed in utero cases

*Basic characteristics*	*CS group* *(n* = *20)*	*Exposed group* *(n* = *303)*	*p*
Prenatal ultrasound—abnormal	1 (5.3%)	0 (0%)	> 0.999
Type of delivery—vaginal	11 (57%)	170 (56%)	> 0.999
Male sex, *n* (%)	13 (65%)	148 (49%)	0.418
Gestational age at birth, weeks, mean ± SD	38.1 ± 3.3	38.7 ± 1.8	0.116
Birth weight, g, mean ± SD	2760 ± 736	3196 ± 517	**0.045**
Microcephaly	6 (30%)	27 (9%)	**0.034**
RPR titers at the time of diagnosis (positive)	20 (100%)	0 (0%)	** < 0.001**
RPR fourfold higher than the maternal test	11 (55%)	0 (0%)	** < 0.001**
Time to negativization of TPHA (months)	5 ± 2.4	6 ± 3.5	*0.490*
Time to negativization of RPR (months)	6.1 ± 3.2	3.4 ± 1.3	***0.046***

p-value statistically significant are in bold

Based on the time of maternal diagnosis and the adequacy of treatment before or during pregnancy, each newborn was classified according to a risk scenario. The comparison between the two variables showed a significant correlation between the severity of the scenario and the administration of the therapy (*p* < 0.001).

Twelve patients (60%) received CS diagnosis in the first month of life, six (30%) within 2 years, while two children (10%) were diagnosed with late CS. Ten babies (50%) were born between 2018 and 2022. This data translates to rates of 2.4 cases × 10^5^ live births between 2010 and 2017 and 5 × 10^5^ live births between 2018 and June 2022.

**Fig. 3:**
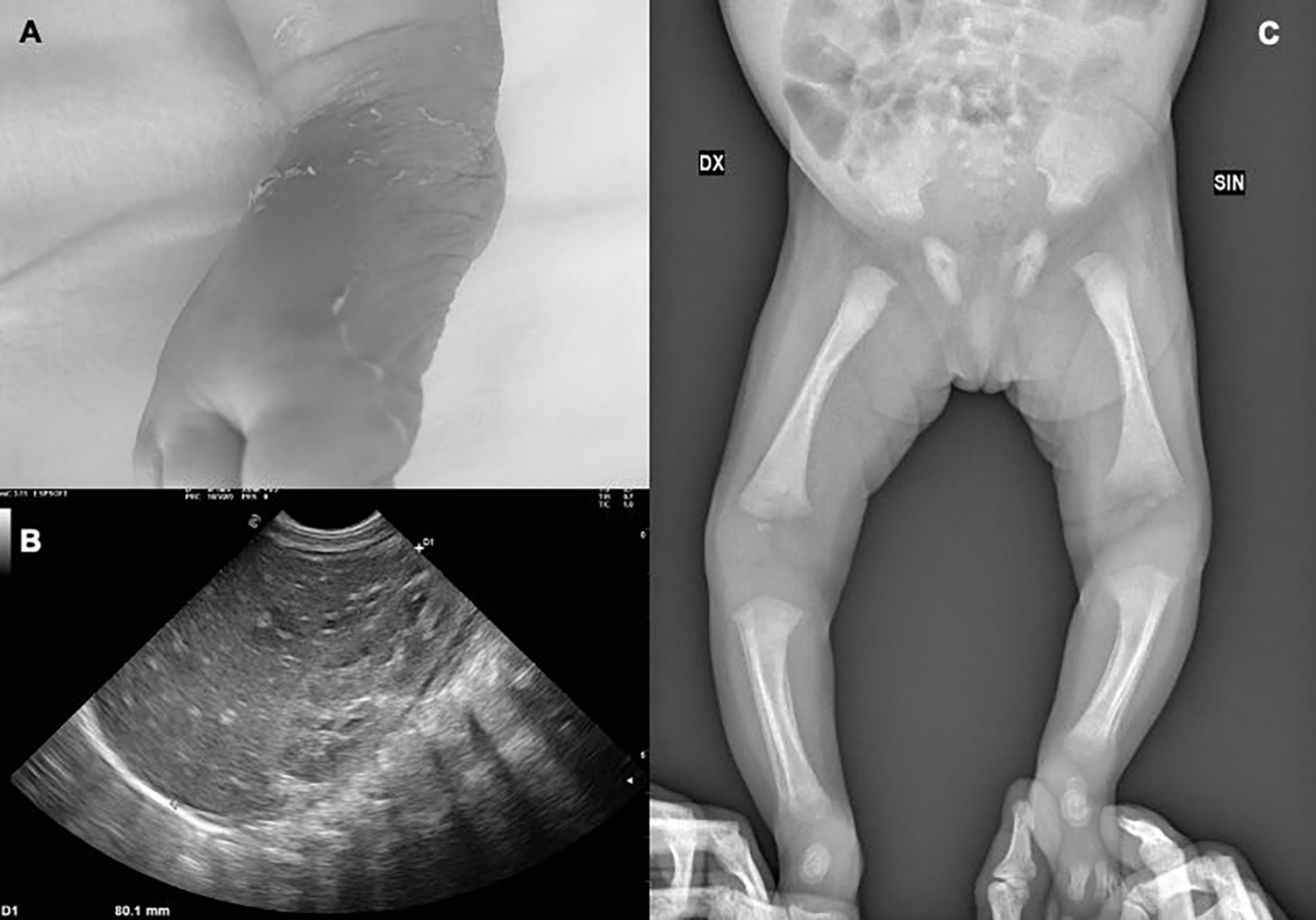
Clinical features of confirmed CS patients. **A** Cutaneous palmar-plantar bullous lesions. **B** Hepatomegaly with inhomogeneous parenchyma. **C** Periostitis and osteitis of tibia

Figure 3 shows some clinical manifestations of CS that were found in 15 patients (75%) but in none of the exposed infants (*p* < *0.001*). The most frequent manifestation (45%) was a maculo-papular rash with bullous evolution. Eight (40%) patients presented clinical signs of periostitis and osteochondritis of the humerus, radius, and ulna, resulting in Parrot’s pseudo paralysis and corresponding radiographic changes. Four infants presented with signs of respiratory distress with need for non-invasive respiratory assistance; however, two of them were preterm, and therefore with additional risk factors. One patient presented with chorioretinal scar without subsequent visual damage. None of the patients experienced hearing loss at birth or later.

Blood chemistry tests revealed severe anemia in seven patients with CS (35%), who then required transfusion of concentrated red blood cells, while thrombocytopenia was observed in three patients. Three children presented with elevated liver transaminase, despite the absence of liver abnormalities on ultrasound examination. On the other hand, hepatosplenomegaly was shown at the examination and ultrasound of the abdomen in four patients (20%), one with comet tail hyperechoic spots, as from cholestasis, confirmed by an increase in direct bilirubin. No blood or abdomen ultrasound abnormalities were found in children who were only exposed.

While all infected patients had positive RPR titers, a fourfold increase was documented in 11 (55%) infants and a twofold increase in one case. Four (20%) mothers had a higher specific titer than their offspring. The remaining mother-infant dyads had equivalent antibody titers. Lumbar puncture with RPR evaluation on CSF was performed on 18/20 children with CS, with a positive antibody titer in 11 children (61% on 18 performed). Conversely, no specific anomalies related to syphilis were highlighted in brain imaging tests (cerebral ultrasound/magnetic resonance). CSF evaluation presented with normal results, for the 112/303 of exposed children who were classified as high-risk. Moreover, all these infants had normal cerebral ultrasound images. Penicillin was administered in 112 exposed patients (using intramuscular administration in 76 cases and intravenous in 36). All infected infants and children were treated with intravenous penicillin, at the recommended dosage for 10 days, except for patients diagnosed with neurosyphilis who received therapy for 14 days. Therapy in the children was more frequent in case of diagnosis in the third trimester or at delivery (OR 3.46; 1.04, 12.3 95% CI; *p* 0.044). Appropriate maternal therapy was protective for the MTCT (β − 1.2; − 1.4, − 1 95% CI; *p* < 0.001).

Patients with CS underwent serological evaluations every 3 months after treatment until 18 months and then twice in a year. Analyzing single factors that could be related with time to negativization of RPR, we found that it was longer in infected children but also in later maternal diagnosis; in primary and secondary maternal stage; in the presence of clinical signs of primary and secondary syphilis; in case of microcephaly; and in the presence of symptoms at birth, high neonatal class of risk, and intravenous therapy. However, time to negativization of RPR was inversely related to number of previous pregnancies, latent maternal stage, and intramuscular therapy (see Supplementary material).

The median time of follow-up was 22 months (9.6–25.5 months). Four children with CS (20%) showed delayed acquisition of the developmental stages but with normal neurological outcome at the end of period of evaluation in two cases, while one child was diagnosed with autism spectrum syndrome and another one with attention-deficit hyperactivity disorder (ADHD). Two patients were admitted to pediatric intensive care unit because of multiorgan failure of which one died. One patient was missed immediately after discharge so data on outcome are not available. None of the exposed children developed any significant problem at the end of evaluation period.

Furthermore, we compared patients enrolled before and after the introduction of free screening for syphilis in the third trimester or peripartum in the absence of previous checks (in 2017), in addition to screening practiced in the first trimester of pregnancy. Until 2017, the number of children referred was 20 per year, which then increased to 62 per year. The rate of late maternal diagnoses did not significantly differ in the two periods (*p* = *0.742*). Moreover, the state of infection in children does not appear to be statistically related to the time of introduction of the screening test in the third trimester of pregnancy (6.3% pre-2017 vs 5.4% post-2017; *p* > *0.999*).

PCR testing in blood was negative for all the patients, in both the CS group and the exposed group.

**Table 3:** Basic maternal features of confirmed CS and exposed in utero cases

*Basic characteristics*	*CS group* *(n* = *20)*	*Exposed group* *(n* = *303)*	*p*
Age, years (median ± DS)	25 ± 7.2	29.9 ± 6	***0.041***
Previous pregnancies, *n* (media)	0.64	1.11	***0.044***
Adequate maternal treatment	0 (0%)	191 (63%)	0.101
Inadequate maternal treatment	1 (5%)	29 (9.6%)	0.101
No maternal treatment	19 (95%)	83 (27%)	** < 0.001**

p-value statistically significant are in bold

### Maternal features

Basic maternal features of confirmed CS and exposed in utero cases are shown in Table 3.Women affected by syphilis were mainly of Italian nationality (206/323, 68%), although among foreigners, 52% (61/117) came from Eastern European countries without differences between mothers of infected children and exposed children (*p* = *0.307*).

Drug abuse was declared in 2.5% (8/323) of the maternal population, with no differences between the two subgroups (*p* = *0.338*). However, there was a statistically significant association with the time of diagnosis of syphilis (*p* = *0.0031*), which occurred later (third trimester or post-pregnancy) in this group than in non-drug addicted women. In 22 mothers with syphilis (6.8%), other concomitant infections with potential vertical transmission were observed (eight with HCV, five with HIV, five with HBV, and four with concomitant syphilis, HIV, and HCV), with no difference between the two subgroups (*p* = *0.755*).

The diagnosis of syphilis was made at the first prenatal screening (first trimester of gestation) in 66% of the population, 14% received the diagnosis at the time of delivery, while 2.6% of the affected mothers discovered syphilis through the appearance of CS symptoms in the affected child or during a subsequent pregnancy as they were not tested during the previous pregnancy. Mothers of CS children received a diagnosis of syphilis at a later stage of pregnancy (86% vs 20%, from third trimester or later; *p* < 0.001). In this group, the infection was defined as primary or secondary in 33% vs 5.9% (*p* = *0.009*).

The only mother of an infected child who received therapy did not have an adequate serological response. The protective action of adequate maternal treatment on children was confirmed by multiple linear regression analysis (β − 1.2; 95% CI − 1.4. − 1.0, *p* < *0.001*).

There was no difference regarding marital status and partner infection between the two subgroups. However, information regarding the diagnosis of syphilis in the partner was poorly documented (36% of cases). From available data, only 19% of partners were identified as positive for treponemal and non-treponemal tests at maternal screening, with a significant correlation between the marital status of the mother and positivity to the paternal evaluation (*p* = 0.006). Of these, 70% received adequate treatment. The paternal diagnosis of infection has a statistically significant correlation (*p* < *0.001*) with the introduction of free screening in the third trimester of pregnancy from 2017.

## Discussion

Our data unravel that CS, although preventable and curable, is still a serious and potentially fatal disease in a European country. In our cohort, one infant died at 2 months of age from multiorgan failure unresponsive to penicillin and aggressive life support therapy, while another presented with respiratory failure which required intensive care and shows mild developmental delay at 4 months of age.

In a 12-year interval, we diagnosed 20 confirmed cases with a rate (3.2 × 10^5^ live births) that is slightly over sixfold the eradication threshold set by the WHO. The clinical features of the infants in our cohort do not differ markedly from what has been reported [4, 9, 20]. Instead, maternal findings partially diverge from the cross-sectional study in 94 Italian hospitals (with an unclear proportion of the national live births) conducted in 2007 [21]. By means of a questionnaire to be completed at delivery for every woman with a positive syphilis serology, Tridapalli et al. reported that 76% of infants with probable (*n* = 51) or possible CS cases (*n* = 76) were born to foreign mothers.

Fifteen years later, we observed a prevalence of Italian women in our population. As in the previous paper, however, these women had little or no antenatal screening despite the introduction in 2017 of an additional free screening for check syphilis during the third trimester of pregnancy. They also had fewer previous pregnancies than the control group. These features point out a higher risk profile that deserves a targeted preventive intervention.

As per the foreign-born mothers, we confirm the preponderance of Eastern European origin. With the possible exception of Bulgaria, official data does not show in this part of the continent a high number of confirmed CS cases [14]. The structure and efficiency of a national CS surveillance program for those countries is not available in the international literature.

True CS eradication will not be reached without full knowledge of the male partner syphilis status. We report significant difficulties in gathering this information possibly due to cultural barriers and personal reticence. However, the positive trend in this respect since 2017 will make parental counseling more rewarding.

The third trimester, free, serological screening available since 2017 to date, was not associated to a decrease of confirmed CS diagnoses. On the other hand, a larger number of exposed infants is being registered, despite a possible decreased access to prenatal care caused by the COVID-19 pandemic. Moreover, even when maternal treatment was absent or inadequate (112 cases), a timely postnatal treatment led to non-infected status in the offspring. We believe that these results constitute an initial success of our area-based prevention program.

We show that an RPR fourfold antibody increase in the infant is not a sensitive feature and that an accurate investigation of maternal status remains the mainstay for a pre-symptomatic CS diagnosis. Previous literature often offers little information on the duration and the quality of the follow up program for CS [9, 10, 22, 23]. We underline the importance of a thorough neonatal follow-up that allowed us to suspect and then confirm 8 CS diagnoses after the neonatal period. This issue has also been emphasized by Kimball et al. [7] who described 67 symptomatic infants diagnosed beyond day of life 28 born in the USA between 2014 and 2018.

During this interval, the CDC also reports a total CS cases increase of 183% which has not been confirmed in European reports. In our area-based cohort, we have witnessed a significant increase over the years of suspected cases to be screened. It remains to be elucidated whether this might be due to the disease resurgence and/or to progress in regional networking against perinatal infections. In our opinion, the latter remains the preferable model for such a delicate public health matter. A dedicated unit has several advantages over the occasional CS diagnosis in any hospital. A SPIU keeps track of patients’ history and medical records, it concentrates specific expertise, participates to national and international registers, and promotes local prevention programs. These factors can both aid new clinical research and increase awareness on CS.

## Conclusions

Fifteen years after a national cross-sectional study, we show that CS is still far from being eradicated in Italy. A specialized health care unit can help in the thorough surveillance of a population and implement preventive medicine.

## Supplementary Information

Below is the link to the electronic supplementary material.

Supplementary file1 (DOCX 16 KB)

## Abbreviations

ABR: Automatic auditory brainstem response
ADHD: Attention-deficit hyperactivity disorder
CDC: Centers for Diseases Control and Prevention
CI: Confidence intervals
CS: Congenital syphilis
CSF: Cerebrospinal fluid
CUS: Cranial ultrasonography
ELISA: Specific enzyme-linked immunosorbent assay
IQR: Interquartile range
MTCT: Mother-to-child transmission
NTT: Non-treponemal test
OR: Odds ratios
PCR: Polymerase chain reaction
RPR: Rapid plasma reagin test
SD: Standard deviation
SPIU: Specialized Perinatal Infection Unit
TEOAE: Transitory evoked otoacoustic emissions
TPHA: *T. pallidum* Hemagglutination test
TT: Treponemal test
WHO: World Health Organization

## References

[1] Tsimis ME, Sheffield JS (2017). Update on syphilis and pregnancy. Birth Defects Research.

[2] Committee on Infectious Diseases AA of P, Kimberlin DW, Barnett ED, Lynfield R, Sawyer MH (2021) Red Book: 2021–2024 Report of the Committee on Infectious Diseases. 10.1542/9781610025782

[3] Schlueter A, Doshi U, Garg B, Hersh AR, Caughey AB (2021) Adverse pregnancy outcomes associated with maternal syphilis infection. J Maternal-Fetal Neonatal Med. 10.1080/14767058.2021.1895740 10.1080/14767058.2021.1895740 33678095

[4] Uku A, Albujasim Z, Dwivedi T, Ladipo Z, Konje JC (2021). Syphilis in pregnancy: the impact of “the Great Imitator”. European Journal of Obstetrics and Gynecology and Reproductive Biology.

[5] Su JR, Brooks LC, Davis DW, Torrone EA, Weinstock HS KML (2016) Congenital syphilis: trends in mortality and morbidity in the United States, 1999 through 2013. Am J Obstet Gynecol 214(3):381.e1–9. 10.1016/j.ajog201510007. Epub 2015 Oct 16 PMID: 26470826; PMCID: PMC6463496 10.1016/j.ajog.2015.10.007 PMC6463496 26470826

[6] Rowe CR, Newberry DM, Jnah AJ (2018). Congenital syphilis: a discussion of epidemiology, diagnosis, management, and nurses role in early identification and treatment. Adv Neonatal Care.

[7] Kimball A, Torrone E, Miele K et al (2018) Morbidity and mortality weekly report missed opportunities for prevention of congenital syphilis-United States. 2018:69(22); available at https://www.cdc.gov/mmwr/mmwr_continuingEducation.html 10.15585/mmwr.mm6922a1 PMC7272112 32497029

[8] Meredith Stephanie, World Health Organization (2007) The Global Elimination of Congenital Syphilis : Rationale and Strategy for Action. World Health Organization

[9] Dai Y, Zhai G, Zhang S, Chen C, Li Z, Shi W (2022) The clinical characteristics and serological outcomes of infants with confirmed or suspected congenital syphilis in Shanghai, China: a hospital-based study. Front Pediatr 10. 10.3389/fped.2022.802071 10.3389/fped.2022.802071 PMC8904424 35281239

[10] Cooper JM, Porter M, Bazan JA, Nicholson LM, Sánchez PJ (2018). Re-emergence of congenital syphilis in Ohio. Pediatric Infectious Disease Journal.

[11] Bowen VB, McDonald R, Grey JA, Kimball A, Torrone EA (2021) High congenital syphilis case counts among U.S. infants born in 2020. New England J Med 385(12):1144–1145. 10.1056/nejmc2111103 10.1056/NEJMc2111103 34525291

[12] Araújo MAL, Esteves ABB, Rocha AFB, da Silva Junior GB, Miranda AE (2021). Factors associated with prematurity in reported cases of congenital syphilis. Rev Saude Publica.

[13] Spiteri G, Unemo M, Mårdh O, Amato-Gauci AJ, The resurgence of syphilis in high-income countries in the,  (2000). a focus on Europe. Epidemiol Infect.

[14] European Centre for Disease Prevention and Control. Syphilis and congenital syphilis in Europe : a review of epidemiological trends (2007–2018) and options for response; available at https://www.ecdc.europa.eu/en/publications-data/syphilis-and-congenital-syphilis-europe-review-epidemiological-trends-2007-2018

[15] Red Book: 2021–2024 Report of the Committee on Infectious Diseases (32nd Edition) ISBN-13: 978-1-61002-521-8

[16] Gruppo Multidisciplinare (2012) Malattie infettive in ostetricia-ginecologia e neonatologia. Percorsi Diagnostico-Assistenziali in Ostetricia-Ginecologia e Neonatologia Sifilide

[17] Zhu L, Gu X, Peng RR (2014). Comparison of the cerebrospinal fluid (CSF) toluidine red unheated serum test and the CSF rapid plasma reagin test with the CSF Venereal Disease Research Laboratory test for diagnosis of neurosyphilis among HIV-negative syphilis patients in China. J Clin Microbiol.

[18] Versiani I, Cabral-Castro MJ, Puccioni-Sohler M (2019). A comparison of nontreponemal tests in cerebrospinal fluid for neurosyphilis diagnosis: equivalent detection of specific antibodies. Arq Neuropsiquiatr.

[19] CDC Treatment guidelines for congenital syphilis; available at https://www.cdc.gov/std/treatment-guidelines/congenital-syphilis.htm

[20] Onesimo R, Buonsenso D, Gioè C, Valetini P (2012) Congenital syphilis: remember to not forget. BMJ Case Rep 2012. 10.1136/bcr.01.2012.5597. PMID: 22669017; PMCID: PMC3369312 10.1136/bcr.01.2012.5597 PMC3369312 22669017

[21] Tridapalli E, Capretti MG, Reggiani MLB et al (2012) Congenital syphilis in Italy: a multicentre study. Arch Dis Childh Fetal Neonatal Ed 97(3). 10.1136/adc.2010.183863 10.1136/adc.2010.183863 20870907

[22] Zammarchi L, Borchi B, Chiappini E (2012). Syphilis in pregnancy in Tuscany, description of a case series from a global health perspective. Journal of Maternal-Fetal and Neonatal Medicine.

[23] Buffolano W, Agnese M, Pizzuti R (2011). Secular trend on congenital infections: insights from Campania region register for perinatal infection, southern Italy. Journal of Maternal-Fetal and Neonatal Medicine.

