# Supplementary material for: Congenital syphilis in the twenty-first century: an area-based study
Source: Eur J Pediatr. 2022 Nov 14;182(1):41–51. doi: 10.1007/s00431-022-04703-5 (PMC9663170; doi:10.1007/s00431-022-04703-5)
Supplement: Supplementary file 1 — Supplementary file1 (DOCX 16 KB) [file 431_2022_4703_MOESM1_ESM.docx]

***Table. Simple linear regression related to time to negativization of RPR (days)***

| **Variable** | **β** | **95% CI** | **p-value** |
| --- | --- | --- | --- |
| Status of infection   - exposed - infected | -  91 | -  56, 125 | <0.001 |
| Number of previous pregnancies | -6.1 | -12, -0.67 | 0.028 |
| Time of maternal positive test   - first trimester - from third trimester | -  25 | -  8.9, 41 | 0.002 |
| Stage in pregnancy   - serological scar - latent - primary - secondary | -  8.6  68  60 | -  -6.6, 24  27,109  23, 96 | <0.001 |
| Clinical signs of primary syphilis   - no - yes | -  70 | -  27, 114 | 0.002 |
| Clinical signs of secondary syphilis   - no - yes | -  45 | -  13, 78 | 0.007 |
| Paternal serological status   - negative - positive | -  25 | -  3.4, 47 | 0.024 |
| Microcephaly   - no - yes | -  25 | -  1.6, 49 | 0.037 |
| Presence of symptoms at birth   - no - yes | -  130 | -  94, 166 | <0.001 |
| Neonatal class of risk   - high probable - improbable - possible - probable | -  -82  -75  -73 | -  -116, -48  -107, -43  -105, -40 | <0.001 |
| therapy in child   - no - yes IV - yes IM | -  28  -4.3 | -  9.3, 48  -20, 12 | 0.007 |
